# The Impact of Microgravity and Hypergravity on Endothelial Cells

**DOI:** 10.1155/2015/434803

**Published:** 2015-01-13

**Authors:** Jeanette A. M. Maier, Francesca Cialdai, Monica Monici, Lucia Morbidelli

**Affiliations:** ^1^Department of Biomedical and Clinical Sciences “L. Sacco”, Università di Milano, Via Gian Battista Grassi 74, 20157 Milan, Italy; ^2^ASAcampus Joint Laboratory, ASA Research Division, Department of Experimental and Clinical Biomedical Sciences “M. Serio”, University of Florence, Viale Pieraccini 6, 50139 Florence, Italy; ^3^Department of Life Sciences, University of Siena, Via A. Moro 2, 53100 Siena, Italy

## Abstract

The endothelial cells (ECs), which line the inner surface of vessels, play a fundamental role in maintaining vascular integrity and tissue homeostasis, since they regulate local blood flow and other physiological processes. ECs are highly sensitive to mechanical stress, including hypergravity and microgravity. Indeed, they undergo morphological and functional changes in response to alterations of gravity. In particular microgravity leads to changes in the production and expression of vasoactive and inflammatory mediators and adhesion molecules, which mainly result from changes in the remodelling of the cytoskeleton and the distribution of caveolae. These molecular modifications finely control cell survival, proliferation, apoptosis, migration, and angiogenesis. This review summarizes the state of the art on how microgravity and hypergravity affect cultured ECs functions and discusses some controversial issues reported in the literature.

## 1. The Endothelium

The concept of endothelium as an inert barrier lining the inner side of blood vessels has been overcome by the finding that the endothelium is a dynamic, heterogeneous, and disseminated organ which orchestrates blood vessel and circulatory functions, thus exerting a critical role for tissue homeostasis. Indeed, the endothelial cells (ECs) possess essential secretory, synthetic, metabolic, and immunologic activities [1, 2].

The endothelium is semipermeable and regulates the transport of various molecules between the blood and underlying interstitial space by expressing specific carriers. ECs also control vascular permeability, especially in microvascular districts. Moreover, ECs importantly contribute to maintaining a nonthrombogenic blood-tissue interface since they release various antithrombotic and fibrinolytic factors as well as molecules that impact on platelets [1, 2].

The endothelium is an immunocompetent organ because it exposes histocompatibility and blood group antigens can be induced to express adhesion molecules for leukocytes and produce cytokines. Finally, a functional relation exists between endothelial and smooth muscle cells, as a consequence of the presence of junctions allowing the passage of electric charges and metabolites, and the production and release of vasoactive mediators [1, 2]. Indeed, ECs finely control vasomotor responses through the production and metabolism of vasoactive molecules acting on smooth muscle cells, as endothelin-1 (ET-1), nitric oxide (NO), and angiotensin II (AngII). They also tightly control smooth muscle cells proliferation [1, 2]. ECs are protagonists in angiogenesis, that is, the formation of new blood vessels from preexisting ones. Angiogenesis involves the most dynamic functions of the endothelium, since it requires the migration of ECs, their ability to degrade the extracellular matrix, their proliferation and differentiation, ultimately leading to functional capillaries [3]. This highly organised process is modulated by the balance between stimulators and inhibitors of angiogenesis.

Vascular endothelium is structurally and functionally heterogeneous [4]. This heterogeneity is detectable at different levels, that is, markers of cell activation, gene expression, responsiveness to growth factors, and antigen composition, and differentiates the behaviour between micro- and macrovascular ECs, as well as between cells isolated from different organs and from different vascular districts of the same organ. In fact, the arteriolar endothelium is different from the venous one, as well as from the micro- and macrovessel derived ECs. The endothelium of the cerebral circulation—which is the main component of the blood-brain barrier to protect the brain from toxic substances—deserves special consideration. It is continuous, has tight junctions, and differs both from fenestrated endothelium, where cells have pores, and from discontinuous endothelium, where cells have intracellular and transcellular discontinuities [2].

ECs are normally quiescent* in vivo* with a turnover rate of approximately once every three years [5]. Most of ECs in the adult have a cell cycle variable from months to years, unless injury to the vessel wall or angiogenesis occurs. Only endothelium from endometrium and corpus luteum has a doubling time of weeks.

ECs act as mechanotransducers, whereby the transmission of external forces induces various cytoskeletal changes and activates second messenger cascades, which, in turn, may act on specific response elements of promoter genes. Therefore, it is not surprising that ECs are sensitive to variations of gravity.

## 2. Methods to Simulate Microgravity and Hypergravity on Earth

Gravity is exerted permanently on organisms which are in constant orientation in the gravity field (static stimulation) and if their orientation is changed with respect to the gravity vector (dynamic stimulation) [6].

The only way to achieve real microgravity is to use parabolic flights, rockets, space crafts, or space labs as available on the International Space Station (ISS). However, the possibility to perform experiments in real microgravity is limited because of high costs and the limited number of missions. On the other hand, the short duration of microgravity conditions achieved by using parabolic flights or rockets limits the studies of many complex and prolonged biological processes. Therefore, many efforts have been made to establish methods to simulate microgravity on Earth. All the devices available, however, mimic only some aspects of real microgravity.

### 2.1. Clinostat

Clinostats are considered reasonably effective ground-based tools for simulating microgravity [7–10] and have been used to study the effects of microgravity [11–19].

The clinostat randomizes motion and theoretically reduces the uniform gravity influence. In the more widely diffuse design, that is, the random positioning machine (RPM), the clinostat consists of an inner chamber containing the samples which rotate clockwise, anticlockwise, vertically, and horizontally. The horizontal and vertical motions are provided by an outer chamber. All the chambers are operated by small motors under computer control. The cells are grown in cell chambers or in flasks filled completely with media, thus diminishing the likelihood of turbulence and shear forces during culture rotation. When using the clinostat to simulate microgravity, the shear stress and vibrations generated by the clinostat must be taken into account. Shear stress can be limited by completely filling the chamber with the culture medium. Parallel controls are necessary to eliminate the effects of vibrations. It is also important to consider the distance of the samples from the centre of the platform, where the maximal reduction of gravity occurs. Another important parameter to monitor is the speed of rotation. It has been verified that the effects of clinostat-determined microgravity are similar to those obtained in space labs [8–19].

### 2.2. Rotating Wall Vessel Bioreactor

This device was developed at NASA's Johnson Space Center to simulate the effects of microgravity on cells in a ground-based culture system. The bioreactor, the rotating wall vessel (RWV)/rotating cell culture system (RCCS) from Synthecon (http://www.synthecon.com/), is a cylindrical vessel that maintains cells in suspension by slow rotation around its horizontal axis with a coaxial tubular silicon membrane for oxygenation. Adherent cells need to be cultured on beads. This system represents a new cell culture technology developed for 3D cultures of different cell types and biotechnological applications. The vessel wall and the medium containing cells bound to microcarrier beads or 3D cultures rotate at the same speed, producing a vector-averaged gravity comparable with that of near-Earth free-fall orbit [20]. Most results obtained using the RWV were confirmed by experiments in real microgravity [12, 21–23].

### 2.3. Magnetic Levitation

This is a relatively novel Earth-based simulation technique used to investigate the biological response to weightlessness. Magnetic levitation takes place when the magnetic force counterbalances the gravitational force. Under this condition, a diamagnetic sample is in a simulated microgravity environment. However, the magnetic field which is generated affects cell behaviour, therefore confounding the effects of simulated microgravity. Mouse osteoblastic MC3T3-E1 cultured in a superconducting magnet for 2 days showed marked alterations of gene expression [24]. Random rotation and magnetic levitation induced similar changes in the actin of A431 cells that were also described in real microgravity [25]. At the moment, however, no studies are available on ECs under magnetic levitation, but they should be fostered as levitation as an alternative to simulate microgravity might yield novel information or confirm previous data, thereby helping in designing successful experiments in real microgravity.

### 2.4. Models to Generate Hypergravity

Variation in gravity exposure is also related to hypergravity, as the one to which the astronauts are transiently exposed during launch and return to Earth. Also military pilots and subjects engaged in certain sports such as motor racing, motorcycling, bobsledding, and the luge experience hypergravity. The comparison among the conditions of microgravity, normogravity (1 ×g), and hypergravity may be helpful to understand the mechanisms underlying the effects of gravitational alterations on endothelial function and to understand what happens when humans quickly pass from hypergravity to microgravity conditions and vice versa.

Centrifuges constructed for research under hypergravity conditions are characterized by high precision control of rpm. Their speed and the angle of inclination of the sample can be regulated to obtain the desired hypergravity in a range from 1 to many g. Centrifuges are also used to perform 1 ×g control experiments on board of the ISS and spacecraft. Studies on endothelial cells in hypergravity are available [12, 26, 27].

## 3. The Effects of Microgravity on ECs

Exposure to microgravity during space missions impacts on various systems. In humans microgravity-induced alterations include bone loss, muscle atrophy, cardiovascular deconditioning, impairment of pulmonary function, and immune response [50, 51]. The cardiovascular system is affected by spaceflight, with changes manifesting as cardiac dysrhythmias, cardiac atrophy, orthostatic intolerance, and reduced aerobic capacity [52]. These changes can cause adaptation problems when astronauts return back to Earth, especially after long-duration spaceflights [53].

Because ECs are key players in the maintenance of vascular integrity, inflammation, and angiogenesis, several studies have been devoted to the mechanisms by which microgravity affects EC functions.

Various reports have indicated that ECs are highly sensitive to microgravity and undergo morphological, functional, and biochemical changes under these conditions [11, 12, 23, 28, 29, 37, 38, 46, 49, 54]. These studies have used a variety of* in vitro* cell models with divergent results. One of the reasons for these discrepancies can be EC heterogeneity or the isolation from different species. Indeed, human, bovine, murine, and porcine endothelial cells have been investigated under gravitational unloading. With concern to human cells, studies are available on human ECs from the umbilical vein (HUVEC), widely considered a model of macrovascular endothelial cells, as well as on human microvascular ECs (HMEC). Moreover, studies have been performed on EA.hy926 cells, a fusion of HUVEC with the lung carcinoma cell line A549 [55]. Although immortalized cell lines offer significant logistical advantages over primary cells in* in vitro* studies, they exhibit important differences when compared to their primary cell counterparts. Indeed, microarrays used for a genome-wide comparison between HUVEC and EA.hy926 in their baseline properties have shown that EA.hy926 cells are useful in studies on genes encoding molecules involved in regulating thrombohemorrhagic features, while they appear to be less suited for studies on the regulation of cell proliferation and apoptosis [56]. Moreover, immortalized endothelial cell lines show different expression pattern of biomarkers when compared to primary cells [57]. The controversial results reported about the response of ECs to microgravity could be due also to the diverse experimental approaches utilized, such as the device simulating microgravity, the duration of exposure to simulated microgravity, and the degree of reduction of the gravity that can be reached operating these devices differently (see above). Nevertheless, altered EC morphology, cell membrane permeability and senescence are documented by spaceflight experiments on cultured endothelium [21, 30, 58].

Several aspects of endothelial behaviour have been studied in simulated and real microgravity.  Table 1 summarizes the published findings.

### 3.1. Migration

Controversy exists on this topic. No significant modulation of cell migration under basal condition and in response to the angiogenic factor hepatocyte growth factor (HGF) was observed in HUVEC as well as in HMEC cultured in the RPM [12, 46]. Shi et al. [39] demonstrated that, after 24 h of exposure to simulated microgravity in a clinostat, HUVEC migration was significantly promoted through the eNOS pathway upregulation by means of PI3K-Akt signalling. On the contrary, the endothelial cell line EA.hy926 in simulated microgravity migrated more than controls [31], while in a study on porcine aortic endothelial cells (PAEC), microgravity modelled by a RPM caused a marked impairment of cell migration induced by serum or the angiogenic factors vascular endothelial growth factor (VEGF) and fibroblast growth factor-2 (FGF-2) [11].

### 3.2. Proliferation and Formation of 3D Structures

Carlsson and Versari, using the RWV and the RPM, respectively, found that the proliferation rate of HUVECs was reversibly increased under simulated microgravity [12, 28]. Also bovine aortic ECs (BAEC) grew faster in the RWV than controls [35]. On the contrary, simulated microgravity inhibited the growth of HMEC and murine microvascular ECs [23, 46]. The results obtained using microvascular EC are reinforced by the* in vivo* finding showing an impairment of angiogenesis in space. Wound healing, in which neovascularization is an early and fundamental step, is retarded in space-flown animal models [59], and the development of vascular channels in a rat fibular osteotomy model is inhibited after flight, as shown by an experiment carried out during a shuttle mission [60].

Also in PAECs a marked impairment of EC responsiveness to angiogenic factors and a reduced ability to proliferate were reported [11]. Using the endothelial cell line EA.hy926, Grimm et al. [43] showed the formation of 3D tubular structures in clinorotation. After two weeks, a subtype of 3D aggregates was observed with a central lumen surrounded by one layer of ECs. These single-layered tubular structures resembled the intimas of blood vessels. Characterization of these tubular structures revealed that they might originate from double-row cell assemblies formed between the fifth and seventh day of culture under simulated microgravity [43].

### 3.3. Apoptosis

Increased apoptosis after culture in the RPM has been observed in PAEC and the endothelial cell line EA.hy926 [11, 40]. In particular, following exposure to simulated hypogravity, PAEC change their morphology and gene expression pattern, triggering proapoptotic signals. The gene expression profile demonstrated the upregulation of p53, FAS-L, and BAX genes, and the concomitant downregulation of the antiapoptotic protein Bcl-2 and proliferation marker PCNA. The induction of apoptosis was accompanied by mitochondrial disassembly, thus suggesting the activation of the mitochondrial intrinsic pathways [11].

In pulmonary HMEC simulated microgravity-induced apoptosis by downregulating the PI3K/Akt pathways and increasing the expression of NF*κ*B [47]. On the contrary, no apoptosis was observed in HUVEC and dermal HMEC cultured for various times in the RWV or in the RPM, and this has been linked to the rapid induction of heat shock protein (hsp)-70 [28, 46, 48]. Indeed, hsp-70 protects endothelial cells from apoptotic stimuli acting downstream of cytochrome c release and upstream of caspase 3.

### 3.4. Alterations of Cytoskeleton and Extracellular Matrix

The cytoskeleton plays a key role in the adaptation to mechanical stress, including alterations of gravity [61, 62]. Therefore, the changes that cytoskeletal components, such as microtubules, undergo in microgravity can be a key to explaining the effects of weightlessness on cells [63, 64].

Carlsson et al. [28] studied actin microfilaments in HUVEC exposed to microgravity simulated by the RWV. In comparison with controls, the cells showed elongated and extended podia, disorganization of actin microfilaments that clustered in the perinuclear area, and decrease in stress fibers. Moreover, after 96 h exposure, actin RNA levels were downregulated and total actin amounts were reduced. The cytoskeletal modifications were reversible upon return to normal growth conditions (1 ×g). The authors speculated that the reduction in actin amount could be an adaptive mechanism to avoid the accumulation of redundant actin fibers. The same results were obtained when the experiment was replicated by using a RPM to model the microgravity conditions [12]. More recently, in HUVEC exposed to mechanical unloading by RPM, Grenon et al. [29] found disorganization of the actin network with clustering of the fibers around the nucleus. Moreover, they observed that caveolin-1 was less associated with the plasma membrane and adopted a perinuclear localization. Thus the authors advanced the hypothesis that disruption of the actin cytoskeleton organization could impair the translocation of caveolin-1 to the caveolae.

After spaceflight (Soyuz TMA-11), readapted HUVEC cells with subsequent passages exhibited persisting changes in the organization of microtubules, with prominent bundles that occupied the peripheral cytoplasm [30].

In a study carried out by Zhang et al. [32] HUVEC activated with TNF-*α* and exposed to microgravity modelled by RWV demonstrated that, after 30 min, depolymerization of F-actin and clustering of ICAM-1 on cell membrane occurred. Moreover, ICAM-1 and VCAM-1 RNA were upregulated. After 24 h, actin fiber rearrangement was initiated, clustering of ICAM-1 became stable, and the mRNAs of ICAM-1 and VCAM-1 returned to levels comparable with the controls. The authors speculated that actin cytoskeleton rearrangement and changes in levels and distribution of surface adhesion molecules could significantly affect transendothelial migration processes.

Grosse et al. [41] studied the effect of parabolic flight on the cytoskeleton of the endothelial cell line EA.hy926. Every parabola (P) included two hypergravity (1.8 g) periods of 20 s, separated by a 22 s microgravity period. After P1, they observed a rearrangement of *β*-tubulin that accumulated around the nucleus. After P31, *β*-tubulin and vimentin were downregulated. Using the EA.hy926 cell line exposed to parabolic flight, Wehland et al. [42] reported that the actin network underwent a drastic rearrangement, mostly affected by vibration.

Grimm et al. [44] studied the walls of tube-like structures spontaneously formed by the endothelial cell line EA.hy926 cultured in a RPM. They found that the walls consisted of single-layered endothelial-like cells which had produced significantly more *β*_1_-integrin, laminin (LM), fibronectin (FN), and *α*-tubulin than controls. Microgravity-induced upregulation of proteins involved in the extracellular matrix building was confirmed in studies carried out by Monici et al. [36] on cultured bovine coronary venular endothelial cells (CVECs) exposed for 72 h to microgravity modelled by a RPM. The authors observed an increase in actin content and impressive production of actin stress fibers, accompanied by the overexpression and clustering of *β*_1_-integrin, 40% increase in LM, 111% increase in FN content, and formation of a tight and intricate network of FN fibrils.

Since FN and LM are strongly involved in the regulation of cell adhesion/migration, their upregulation and altered networking, together with the changes in actin and integrin patterns induced the authors to hypothesize that the exposure to microgravity causes a dysregulation in cell motility and adhesion to the substrate.

In summary, all of the studies carried out so far demonstrated that microgravity strongly affects cytoskeleton organization and induces a rearrangement of the actin network with clustering of the fibers in the perinuclear area. A similar behaviour has been observed also analysing the microtubule network. Moreover, clustering of adhesion molecules on the plasma membrane and overexpression of proteins of the extracellular matrix have been reported by some authors.

The results are less consistent when considering the expression of cytoskeleton proteins or their RNA. Probably the discrepancies are due to differences in experimental models (different cell populations), protocols, and analytical procedures.

However, it is widely accepted that the microgravity-induced changes in the cytoskeleton can strongly affect the behaviour of endothelial cells in terms of adhesion, migration, and production of extracellular matrix and can interfere with other processes such as translocation of molecules inside the cells, transendothelial migration, and even inflammation and angiogenesis.

### 3.5. Synthesis of Vasoactive Molecules

The levels of vasoactive molecules, such as NO, and ET-1 are modified under microgravity conditions, which also indicates that microgravity may influence both hemodynamic changes and angiogenesis [33, 37]. In particular, HUVEC and HMEC exposed to simulated microgravity using RWV and RPM produce more NO than controls as the result of increased levels of endothelial-nitric oxide synthase (e-NOS) [12], which correlates with the increase of caveolins [26, 29]. In particular, Grenon et al. suggested that the alterations in NO production are mediated by changes in the cytoskeleton detected in all the endothelial types studied [29]. Wang et al. [34] explained the increased amounts of NO in HUVEC after 24 h in simulated microgravity as the results of the upregulation of inducible NOS through a mechanism dependent on the suppression of the activity of the transcription factor AP-1. Also in BAEC NO production was increased [35].

In the endothelial cell line EA.hy926, a reduced release of ET-1 and VEGF was reported [37], while the production of NO was increased via the iNOS-cGMP-PKG pathway [39, 45]. If confirmed* in vivo* in space, these results might, in part, explain the hemodynamic changes and the redistribution of blood flows induced by microgravity.

### 3.6. Genomic and Proteomic Analysis

Microgravity affects several molecular features of ECs markedly modulating gene expression. In HUVEC cultured in the RPM, the secretome was evaluated by a 2D proteomic approach [33]. The proangiogenic factor FGF-2 and the proinflammatory cytokines interleukin-1 (IL-1) and IL-8 were decreased in simulated microgravity, whereas two chemokines involved in leukocyte recruitment, Rantes and Eotaxin, were increased [33]. The unprecedented gene profile analysis on HUVEC cultured on the ISS for 10 days was performed by Versari et al. [21]. 1023 genes were significantly modulated, the majority of which are involved in cell adhesion, oxidative phosphorylation, stress responses, cell cycle, and apoptosis, thioredoxin-interacting protein being the most upregulated. Briefly, in cultured HUVEC, real microgravity affects the same molecular machinery which senses alterations of flow and generates a prooxidative environment that alters endothelial function and promotes senescence [21]. Similar conclusions were reached by Kapitonova et al. [30, 58], who described premature senescence in space-flown HUVEC. By accelerating some aspects of senescence, microgravity offers a big challenge to study the mechanisms implicated in the onset of aging.

Looking at the endothelial cell line EA.hy926, a short term lack of gravity (22 s) generated by parabolic flights significantly influences the signalling pathways [41]. When these cells are cultured for various times from 4 to 72 h on the RPM, a number of proteins of the extracellular matrix implicated in apoptosis are modulated when compared to control cells [40]. In the RPM some EA.hy926 cells form tube-like 3D aggregates, while others continue to grow adherently. 3D aggregates and adherent cells were analyzed by gene array and PCR techniques and compared to controls [38]. 1625 differentially expressed genes were identified and, in particular, the levels of expression of 27 genes changed at least 4-fold in RPM-cultured cells when compared to controls. These genes code for angiogenic factors and proteins implicated in signal transduction, cell adhesion, membrane transport, or enzymes. Fifteen of them, with IL-8 and von Willebrand factor being the most affected, showed linkages to genes of 20 proteins that are important in the maintenance of cell structure and in angiogenesis.

EA.hy926 cell line and human dermal microvascular ECs (HMVECs) were then compared after culture on the RPM for 5 and 7 days [54]. A total of 1175 types of proteins were found in EA.hy926 cells and 846 in HMVECs, 584 of which were common and included metabolic enzymes, structure-related and stress proteins. This proteomic study also highlights that HMVECs develop tube-like 3D structures faster than EA.hy926 possibly through a transient augmentation of ribosomal proteins during the 3D assembling of ECs.

## 4. The Effects of Hypergravity on ECs

A summary of published data on endothelial cell behaviour is reported in Table 2. HUVECs exposed to hypergravity (3 ×g) for 24–48 h showed inhibition of cell growth but unaltered apoptosis, increased COX-2, eNOS, and Cav-1, suggesting a possible role of caveolae in mechanotransduction. Also an increased synthesis of PGI2 and NO, which are also proangiogenic, was observed. However, surprisingly, the formation of capillary-like structure was inhibited [65]. Versari et al. [12], studying the same cells exposed to 3.5 ×g, found increased NO production, enhanced cell migration, but no effects on proliferation. Moreover, altered distribution of actin fibers without modifications of the total amounts of actin was detected [12]. In the same conditions, HUVEC showed a time-dependent decrease in occludin correlating with an increase in paracellular permeability and a decrease in transendothelial electrical resistance, indicating a decrease in EC barrier function [66, 67], with exactly opposing results in BAEC cultured under hypogravity in RWV where increased barrier properties were detected [35].

Koyama et al. [69] reported that, after a few minutes of exposure to 3 ×g in a centrifuge, BAECs showed actin reorganization via Rho activation and FAK phosphorylation, increased cell proliferation, and ATP release. A daily exposure of 1-2 h repeated for 5 consecutive days promoted cell migration. Wehland et al. [42] investigated short term (s) effects of hypergravity (1.8 ×g) on EA.hy926 cells and found that the cells were weakly affected by loading in the conditions used for the experiment. On the contrary, short term effects of microgravity were much more evident.

In order to evaluate these results two considerations have to be made.

1. Very different protocols and parameters have been used for EC exposure to hypergravity: continuous versus discontinuous exposure, different g force value, and exposure times ranging from minutes to days.
2. ECs, both derived from the microvasculature and macrocirculation, are very sensitive to mechanical stress. It should be underscored that, in physiological conditions, the quality and intensity of mechanical stimulation to which the endothelium is exposed depend on the vascular district.

Following the latter consideration, we hypothesized that EC response to gravitational alteration could depend on the district from which the cell population derives and could be different in cells derived from macro- or microcirculation. To verify this hypothesis, we studied and compared the behaviour of coronary venular endothelial cells (CVEC) [27] and BAEC [68] exposed to 10 ×g for 5 periods of 10 minutes each spaced with four recovery periods of the same duration. Following exposure, both the cell types showed similar changes in cytoskeleton organization and *α*v*β*3 integrin distribution. The peripheral ring of actin microfilaments was substituted by trans-cytoplasmic stress fibers, microtubules, and intermediate filaments gathered in the perinuclear area, focal contacts in the protruding lamellipodia disappeared, and *α*v*β*3 integrin molecules clustered in the central body of the cells. Both in CVEC and in BAEC the expression of the cytoskeletal proteins *β*-actin and vimentin increased. In BAEC the transcripts for the matrix proteins LM and FN decreased. In both the cell types exposure to hypergravity decreased the transcription of genes encoding for the proapoptotic factors Fas and FasL, Bcl-XL [27, 68].

Cell energy metabolism, assessed by autofluorescence spectroscopy and imaging, did not change significantly in BAECs. On the contrary, CVECs exposed to hypergravity showed an increase of the anaerobic metabolism, in comparison with 1 ×g controls [27].

The phenotypic expression of molecules involved in inflammation and angiogenesis such as eNOS, FGF-2, and COX-2, which is not expressed in basal conditions, did not significantly change as assessed by immunofluorescence microscopy in CVECs. Nevertheless, in BAECs the expression of* COX-2* and other genes controlling the calibre of the vessels, that is, renin, ET processing enzyme, and inflammation, such as TNF*α* and its receptor CD40, P and E selectins, CD54, was downregulated. Briefly, hypergravity does not seem to affect significantly the survival of both macro- and microvascular ECs. However, significant changes have been observed in cytoskeleton and integrin distribution in all the ECs studied, and changes in cell energy metabolism have been observed only in CVECs, while the downregulation of some genes involved in inflammation and vasoconstriction has been found only in BAECs. Considering the expression of growth modulators, hypergravity increased VEGF expression while it decreased a series of interleukins acting as inhibitors of EC proliferation [27, 68]. These results are consistent with the hypothesis that the EC response to gravitational alterations depends, at least in part, on the vascular district from which the cells are derived.

## 5. Concluding Remarks

The effects of simulated gravity changes on endothelial cells described in various papers are rather discordant but all converge in the indication that endothelial behaviour is significantly altered (Table 3). Briefly, from studies on different types of ECs exposed to simulated microgravity we can summarize the following.

- Impact on cell proliferation and survival: all the studies indicate alterations of cell proliferation. Only HUVEC and BAEC have been reproducibly found to proliferate faster in microgravity than controls. Microvascular EC and other endothelial cells are growth inhibited or induced to apoptosis.
- Impact on NO synthesis: most studies agree on the increased production of NO through the modulation of NOS isoforms.
- Impact on cytoskeleton: all the studies described important cytoskeletal remodelling in all the different EC analyzed.
- Impact on gene expression: no doubt exists about the profound modifications of gene expression by exposure to simulated or real microgravity.

The impact of hypergravity on ECs is less defined. Due to the different experimental approaches adopted on various cell types the findings are not consistent and deserve further consideration.

The effects of gravitational forces on mechanotransduction in ECs responses have been the matter of only a few investigations and remain largely unknown. The plausible mechanosensing targets for gravity changes appear to be the cytoskeletal structure and particularly caveolae [26, 29, 65].

In conclusion, because (i) endothelial cells are crucial for the integrity of the vessel wall and (ii) vessels are responsible for the homeostasis of all the tissues, it is pivotal to continue studies on this topic since the modulation of endothelial functions can contribute to cardiovascular deconditioning and other disorders observed in space, from bone loss to muscle atrophy. However, it would be recommended to clearly define the experimental models to use. A clear cut definition of endothelial cell models to be used and the conditions to model gravity need to be standardized.

## Figures and Tables

**Table 1:** The effects of real or simulated microgravity on different endothelial cell types.

Experimental model	Experimental conditions	Effects	Authors
Primary human umbilical vein ECs (HUVEC)	Rotating wall vessel (RWV) Random positioning machine (RPM) 48 or 96 h	Growth stimulation ↑ NO production Actin remodelling ↓ Actin	Versari et al., 2007 [12]
	Spaceflight (Progress 40P mission) 10 d	↑ Thioredoxin-interacting protein ↓ hsp-70 and 90 ↑ secretion of IL-1*α* and IL-1*β* Ion channels (TPCN1, KCNG2, KCNJ14, KCNG1, KCNT1, TRPM1, CLCN4, CLCA2), mitochondrial oxidative phosphorylation, and focal adhesion were widely affected	Versari et al., 2013 [21]
	RWV 72 h or 96 h	↑ PGI2 and NO	Carlsson et al., 2002 [22]
	RPM 24–48 h	↑ NO ↑ Cav-1 phosphorylation (Tyr 14)	Spisni et al., 2006 [26]
	RWV 4, 24, 48, 96, 144 h	↑↑ hsp70 ↓ IL-1*α* Remodelling of cytoskeleton ↓ actin	Carlsson et al., 2003 [28]
	RPM 24 h	↑ eNOS, Cav-1 and -2 ↓ of the length and width of the cells ↓ ICAM-1, VCAM-1, E-selectin, and IL-6 and TNF-*α*	Grenon et al., 2013 [29]
	Spaceflight 12 d	Cytoskeletal damage ↑ cell membrane permeability In readapted cells: persisting cytoskeletal changes ↓ metabolism and cell growth	Kapitonova et al., 2012 [30]
	2D-Clinostat (developed by China Astronaut Research and Training Center) 30 rpm, 24 h	↑ HUVEC tube formation and migration ↓ number of caveolae in the membrane ↑ eNOS activity by phosphorylation of Akt and eNOS	Siamwala et al., 2010 [31]
	RWV 5 min, 30 min, 1 h, and 24 h	↑ ICAM-1 expression Depolymerization of F-actin and clustering of ICAM-1 on cell membrane (short term) Actin fiber rearrangement and stable clustering of ICAM-1 (after 24 h) ↑ ICAM-1 and VCAM-1 RNA after 30 min	Zhang et al., 2010 [32]
	RPM 96 h	Alteration of proteins regulating cytoskeleton assembly ↓ IL-1*α*, IL-8, and bFGF ↑ chemokines Rantes and Eotaxin, involved in leukocytes recruitment	Griffoni et al., 2011 [33]
	RPM 24 h	↑ iNOS by a mechanism dependent on suppression of AP-1	Wang et al., 2009 [34]

Bovine aortic ECs (BAEC)	RWV for up to 30 d	Growth stimulation ↑ NO Production of NO dependent on the RWV rotation rate: 73% increase at 8 rpm, 262% increase at 15 rpm, and 500% increase at 20 rpm	Sanford et al., 2002 [35]

Porcine aortic ECs (PAEC)	RPM 72 h	↑ proapoptotic genes (p53, FAS-L, BAX) ↓ antiapoptotic genes (Bcl-2) Dissolution of mitochondrial membrane integrity Impairment of cell responsiveness to exogenous stimuli	Morbidelli et al., 2005 [11]

Bovine coronary venular ECs (CVEC)	RPM 72 h	↑ Fibronectin (formation of intricate network of FN fibers) ↑ Laminin ↑ *β*-Actin (formation of stress fibers) ↑ *αβ*-Integrin (formation of clusters)	Monici et al., 2011 [36]

Human EC line EA.hy926	RPM 10 days	↑ Caspase-3, Bax, and Bcl-2 ↑ collagen types I and III Alterations of the cytoskeletal *α*- and *β*-tubulins and F-actin ↓ brain-derived neurotrophic factor, platelet tissue factor, VEGF, and ET-1	Infanger et al., 2007 [37]
	RPM 7 days	Modulation of genes encoding for signal transduction and angiogenic factors, cell adhesion, membrane transport proteins, or enzymes involved in serine biosynthesis	Ma et al., 2013 [38]
	RPM 2 h	↑ cellular migration ↑ filopodia and lamellipodia Actin rearrangements ↑ NO	Shi et al., 2012 [39]
	RPM 4, 12, 24, 48, and 72 h	↑ extracellular matrix (ECM) proteins Alteration in cytoskeletal components ↑ expression of ECM proteins (collagen type I, fibronectin, osteopontin, laminin) and flk-1 protein Morphological and biochemical signs of apoptosis after 4 h, further increasing after 72 h	Infanger et al., 2006 [40]
	Parabolic flight (22 s microgravity, 1.8 xg 2 periods of 20 s)	*Parabolic flight* ↓ COL4A5, COL8A1, ITGA6, ITGA10, and ITGB3 mRNAs after P1 (first parabolas) ↑ EDN1 and TNFRSF12A mRNAs after P1 ↓ ADAM19, CARD8, CD40, GSN, PRKCA mRNAs ↑ PRKAA1 (AMPK*α*1) mRNAs cytoplasmic rearrangement ↑↑ ABL2 after P1 and P31	Grosse et al., 2012 [41]
	Parabolic flight (22 s microgravity, 1.8 xg 2 periods of 20 s)	*Parabolic flight* Actin network rearrangement ↑ CCNA2, CCND1, CDC6, CDKN1A, EZR, MSN, OPN, VEGFA, CASP3, CASP8, ANXA1, ANXA2, and BIRC5 ↓ FLK1 ↑ EZR, MSN, OPN, ANXA2, and BIRC5 after 31P	Wehland et al., 2013 [42]
	RPM 7, 14, 21, and 28 d	Different responsiveness to VEGF and bFGF added exogenously Altered gene and protein expression of phosphokinase A catalytic subunit, phosphokinase C alpha, and ERK-1 and 2 ↓ VEGF, bFGF, soluble TNFRSF5, TNFSF5, ICAM-1, TNFR 2, IL-18, complement C3, and von Willebrand factor	Grimm et al., 2010 [43]
	RPM 7 and 28 d	Delayed 3D cell growth; ↑ beta(1)-integrin, laminin, fibronectin, *α*-tubulin in tube-like structures after 4 weeks of culturing	Grimm et al., 2009 [44]

Human EC line EA.hy926 Bovine lung microvascular ECS Bovine pulmonary aortic ECs Porcine ventricular endocardial ECs	RPM 2 h	Results indicate that iNOS is a molecular switch for the effects of microgravity on different kinds of endothelial cells ↑ angiogenesis via the cyclic guanosine monophosphate (cGMP-) PKG dependent pathway	Siamwala et al., 2010 [45]

Human dermal microvascular cells (HMEC)	RWV, RPM 48 or 96 or 168 h	↑ TIMP-2 ↑ NO ↓ proteasome activity	Mariotti and Maier, 2008 [46]

Murine lung capillary ECs (1G11 cells)	RWV 72 h	↓ endothelial growth ↑ p21 ↓ IL-6 ↑ eNOS and NO	Cotrupi et al., 2005 [23]

Human pulmonary microvascular ECs (HPMECs)	MG-3 clinostat (developed by the Institute of Biophysics Chinese Academy of Sciences)	↑ apoptosis ↓ PI3K/Akt pathway ↑ NF-*κ*B and depolymerization of F-actin	Kang et al., 2011 [47]

Human and bovine microvascular ECs	RWV 96 h	↑ hsp70 in cells which maintained the capability to proliferate in microgravity	Cotrupi and Maier 2004 [48]

Cocultures of endothelial monolayers, human lymphocytes, immune cells, and myeloleucemic (K-560) cells	Spaceflight (ISS)	↑ adhesion of PMA-activated lymphocytes Retained ability of immune cells to contact, recognize, and destroy oncogenic cells *in vitro *	Buravkova et al., 2005 [49]

Legend: ↑, increased; ↓, decreased.

**Table 2:** The effects of hypergravity conditions on different endothelial cell types.

Experimental model	Experimental conditions	Effects	Authors
Primary human umbilical vein ECs (HUVEC)	Hypergravity conditions (generated by a MidiCAR centrifuge at 3.5 xg) for 24–48 h	↑ migration ↑ NO Altered distribution of actin fibers	Versari et al., 2007 [12]
	Hypergravity conditions (generated by a centrifuge at 3 xg) for 24–48 h	↑ cav-1 ↑ distribution of caveolae in the cell interior ↑ COX-2, NO, and PGI2 production ↓ angiogenesis (through a pathway not involving apoptosis)	Spisni et al., 2003 [65]
	Liftoff simulation by centrifuge (7.5 min simulation of the pattern of g forces experienced during liftoff of the NASA space shuttle)	↓ MAPK phosphorylation ↑ occludin expression	Sumanasekera et al., 2006 [66]
	Liftoff simulation by centrifuge (7.5 min simulation of the pattern of g forces experienced during liftoff of the NASA space shuttle)	↑ Paracellular permeability ↓ Occludin ↓ Transendothelial electrical resistance ↓ MAPK activation ↓ EC barrier function	Sumanasekera et al., 2007 [67]

Bovine aortic ECs (BAEC)	Hypergravity (thermostated 3-18K Sigma Zentrifugen, 5 periods of 10 min exposure to 10 xg spaced with 10 min at 1 xg)	Modified integrin distribution Reorganization of cytoskeletal network ↓ genes controlling vasoconstriction and inflammation ↓ Proapoptotic signals	Morbidelli et al., 2009 [68]
	Hypergravity (3 xg) applied by low speed centrifuge	↑ ATP release ↑ actin reorganization via RhoA activation and FAK phosphorylation ↑ cell proliferation and migration	Koyama et al., 2009 [69]

Bovine coronary venular ECs (CVEC)	Hypergravity (thermostated 3-18K Sigma Zentrifugen, 5 periods of 10 min exposure to 10 xg spaced with 10 min at 1 xg)	↓ proapoptotic genes (FADD, Fas, Fas-L) ↑ antiapoptotic gene NF*κ*BChange in cytoskeleton organization Alteration of cell energy metabolism	Monici et al., 2006 [27]

Human EC line EA.hy926	Hypergravity Experiments (MuSIC, DLR, Cologne, Germany centrifuge 1.8 xg) Vibration experiments (Vibraplex vibration platform frequency range 0.2–14 kHz)	↓ CARD8, NOS3, VASH1, SERPINH1 (all P1), CAV2, ADAM19, TNFRSF12A, CD40, and ITGA6 (P31) mRNAs No significant changes on gene expression and morphology of the cells	Grosse et al., 2012 [41]
	Hypergravity Experiments (MuSIC, DLR, Cologne, Germany centrifuge 1.8 xg) Vibration experiments (Vibraplex vibration platform frequency range 0.2–14 kHz)	↓ Pan-actin, tubulin, and Moesin ↓ gene expression of CCND1, MSN, RDX, OPN, BIRC5, and ACTB ↑ Pan-actin, tubulin, and ezrin ↓ *β*-Actin and Moesin ↓ ACTB, CCND1, CDC6, CDKN1A, VEGFA, FLK-1, EZR, ITBG1, OPN, CASP3, CASP8, ANXA2, and BIRC5	Wehland et al., 2013 [42]

Legend: ↑, increased; ↓, decreased.

**Table 3:** Summary of the principal parameters influenced by simulated microgravity and hypergravity in different types of ECs.

	Microgravity					Hypergravity			
	Endothelial cell line EA.hy926	Dermal HMEC	HUVEC	PAEC	BAEC	Endothelial cell line EA.hy926	HUVEC	CVEC	BAEC
Migration	↓	=	=/↑	↓	ND	ND	↑	ND	↑
Proliferation	ND	↓	↑	↓	↑	ND (=/↑)	=	ND	↑
Apoptosis	↑	=	=	↑	=	=/↓	=	=	=
NO synthesis	↑	↑	↑	ND	↑	ND	↑	ND	ND
Cytoskeletal rearrangements	+++	+++	+++	+++	+++	+++	++	+++	+++

Legend: ↑, increased; ↓, decreased; =, no change; ND, not determined; ++ and +++, highly and strongly upregulated.
